# Emerging Insights into Postoperative Neurocognitive Disorders: The Role of Signaling Across the Gut-Brain Axis

**DOI:** 10.1007/s12035-024-04228-y

**Published:** 2024-05-27

**Authors:** Wanqiu Yu, Zhaoqiong Zhu, Fushan Tang

**Affiliations:** 1 Department of Anesthesiology, Affiliated Hospital of Zunyi Medical University, Zunyi, 563003 China; 2 Early Clinical Research Ward, Affiliated Hospital of Zunyi Medical University, Zunyi, 563003 China; 3 Department of Clinical Pharmacy, Key Laboratory of Basic Pharmacology of Guizhou Province, School of Pharmacy, Zunyi Medical University, Zunyi, 563006 China

**Keywords:** Postoperative neurocognitive disorders, Neuroinflammation, Gut microbiota, Intestinal dysbacteriosis, Surgery, Anesthesia

## Abstract

The pathophysiological regulatory mechanisms in postoperative neurocognitive disorders (PNCDs) are intricately complex. Currently, the pathogenesis of PNCDs has not been fully elucidated. The mechanism involved may include a variety of factors, such as neuroinflammation, oxidative stress, and neuroendocrine dysregulation. Research into the gut microbiota-induced regulations on brain functions is increasingly becoming a focal point of exploration. Emerging evidence has shown that intestinal bacteria may play an essential role in maintaining the homeostasis of various physiological systems and regulating disease occurrence. Recent studies have confirmed the association of the gut-brain axis with central nervous system diseases. However, the regulatory effects of this axis in the pathogenesis of PNCDs remain unclear. Therefore, this paper intends to review the bidirectional signaling and mechanism of the gut-brain axis in PNCDs, summarize the latest research progress, and discuss the possible mechanism of intestinal bacteria affecting nervous system diseases. This review is aimed at providing a scientific reference for predicting the clinical risk of PNCD patients and identifying early diagnostic markers and prevention targets.

## Introduction

PNCDs are a common neurological complication that occurs in elderly patients after anesthesia and/or surgery. It is mainly manifested as a decline in cognitive, learning, memory, social, and language thinking abilities and is even accompanied by personality and emotional changes [1]. With the advent of an aging society and the expansion of the upper age limit for indications for surgery/anesthesia, the incidence of PNCDs increased significantly, which seriously impaired the quality of life of patients and increased the burden on families and society [2]. Until now, studies on the pathogenesis and prevention measures of PNCDs are still insufficient [3]. Therefore, exploring the etiology and pathogenesis of PNCDs may provide a theoretical foundation for the prevention and treatment of PNCDs.

The gut microbiota represents a symbiotic microbial community within the host organism, playing a crucial regulatory role in maintaining the health equilibrium and the onset of diseases. A growing body of clinical and animal research has demonstrated that bacteria possess the ability to regulate brain function through bidirectional signaling within the gut-brain axis. Simultaneously, alterations in brain function also impact the gut microbiome via this pathway [4]. Recent studies have shown that intestinal bacteria influence the progression of central nervous system diseases such as Alzheimer’s disease [5], depression and anxiety [6], stroke [7], Parkinson’s disease [8], and multiple sclerosis through the gut-brain axis [9]. It can also affect patients’ postoperative pain and neurocognitive function through remote regulatory pathways such as neurotransmitters, bacterial metabolites, the immune system, and the endocrine system [10, 11]. Importantly, due to the readily targetable nature of the gut microbiota, it was also well recognized as a potential target with practical value in the prevention and treatment of PNCDs.

## Gut Microbiota

Intestinal bacteria constitute a symbiotic microbial community in the host, which plays a critical role in maintaining physiological homeostasis and influencing the occurrence of diseases. The human gut harbors the most abundant bacterial population within the body, hosting over 1000 species of microorganisms, including *Bacteroides*, *Firmicutes*, *Actinomyces*, and *Proteobacteria*. Whether in humans or animals, intestinal bacteria are associated with gastrointestinal diseases and neurological disorders. The gut microbiota can exert complex regulatory effects on multiple systems, including the nervous, immune, endocrine, and metabolic systems, thereby influencing central nervous system signaling and inflammatory responses [6, 12]. Previous studies have found that two types of bacteria in intestinal bacteria, namely, *Ruminococcus* and *Trichospirochaeta*, may be involved in the pathogenesis of postoperative learning and memory disorders [13]. An increase in the abundance of *Proteobacteria* may indicate dysbiosis of the gut microbiota and the risk of disease, potentially leading to chronic inflammation and immune stress responses in the body. Chronic inflammation and immune stress, in turn, may further affect the morphology and function of neurons in the host, impairing cognitive function [14].

### Gut Microbiota Dysbiosis

The structure of the gut microbiota is dynamically evolving, subject to changes influenced by factors such as aging, variations in external environmental conditions, alterations in daily dietary intake, and medication stimuli. Studies have shown a significant difference between the intestinal bacteria of the elderly and young individuals, suggesting that aging may impact the diversity of gut microbiota structure. The ecological structure of the gut microbiota is indispensable for maintaining normal immune function. Therefore, gut microbial diversity may be crucial in sustaining host health [15]. Under normal circumstances, a steady state is maintained between the gut microbiota and the immune system. However, antibiotic usage, high-sugar diets, and chronic stress can impact the gut microbial community. These stimuli disrupt the delicate balance within the microbial community, leading to an increase in harmful bacteria and a decrease in beneficial bacteria within the gut. Lederer et al. found that the number of intestinal bacteria after gastrointestinal surgery decreased to varying degrees, increasing the proportion of potentially pathogenic bacteria. At the same time, the proportion of probiotics such as *Lactobacillus* and *Bifidobacterium* decreased [16]. In addition, in vivo studies have shown that exposure to narcotic drugs causes changes in the composition of the gut microbiota in the host, which last for days or even long term. Serbanescu et al. found that exposure to isoflurane for 4 h resulted in a significant decrease in intestinal bacterial diversity in mice. The abundance of *Firmicutes* decreased at both 24 h and 7 days post-anesthesia, while several commensal bacteria, including *Clostridia*, were completely absent [17]. The anesthetic sevoflurane also alters gut microbiota and related metabolites in the host. Following inhalation of sevoflurane, those treated mice exhibit significant changes in gut microbiota as early as the first day, with a notable decrease in abundance and diversity observed by the seventh day [18, 19].

### The Intestinal Barrier

The intestinal barrier serves as a critical defense line between the body and the external environment. While facilitating digestion and nutrient absorption, it also acts as a barrier, isolating external bacteria and toxic substances. The intestinal barrier comprises multiple layers. Its outermost barrier is composed of mucus on the surface of the intestinal mucosa and intestinal epithelial cells, which regulate intestinal permeability. The intestinal mucus prevents direct contact between epithelial cells and intestinal bacteria or toxic metabolites. At the same time, it provides nutrients for the microbiota residing in the gut, directly influencing the abundance of colonizing bacteria [20, 21]. Intestinal epithelial cells are a layer of tightly arranged cells, including goblet cells, neuroendocrine cells, Pan’s cells, cluster cells, and M cells, which maintain the integrity of intestinal epithelium through tight-junction proteins and other substances [22, 23]. Intestinal epithelial cells could be regulated by factors including intestinal bacterial metabolites, intestinal nerves, and cytokines, thus facilitating the prevention of external invasion and achieving barrier function [24]. In pathological conditions, harmful substances cross the outer intestinal barrier and eventually reach the gut vascular barrier (GVB). This is the last line of defense between the intestinal lumen and systemic circulation, composed of vascular endothelial cells, enteric glial cells, and pericytes, maintaining the integrity of the intestinal vascular barrier. Its functions include transporting nutrients from the intestinal lumen to the systemic circulation for utilization and preventing bacteria and their toxic products from entering the bloodstream [25].

### Intestinal Barrier Dysfunction: Leaky Gut

The intestinal barrier plays an indispensable role in maintaining a healthy homeostasis of the host body. Under normal conditions, it protects the host body from foreign harmful substances through the complementary structure of various layers and further regulates the exchange of substances inside and outside this barrier. The breakdown of the intestinal barrier function can lead to diseases of the digestive system and beyond, and this pathological condition is called "Leaky gut," reflecting increased intestinal permeability. Potential pathological causes may include chronic stress, constipation, and overuse of antibiotics. When the integrity of the outer mucus layer and epithelial cells of the intestine is compromised, leading to increased permeability of the intestinal barrier, bacteria, and bacterial metabolites, those harmful metabolic products from the gut lumen can enter the circulation and disseminate to distant organs. During this process, the host immune system identifies harmful substances in the bloodstream, triggering an inflammatory response, which can lead to acute or chronic inflammation and various diseases [25]. Research has established a well-described link between the onset of certain pathological conditions and the dysfunction of gut barrier function, including disorders such as bloating, enteritis, autoimmune diseases, depression, and inflammatory bowel diseases [26]. Additionally, in vivo studies have linked gut barrier dysfunction with neurological conditions such as Parkinson’s [27, 28], Alzheimer’s [5], amyotrophic lateral sclerosis [29], and cognitive decline associated with aging [30].

## Postoperative Neurocognitive Disorders

**Fig. 1:**
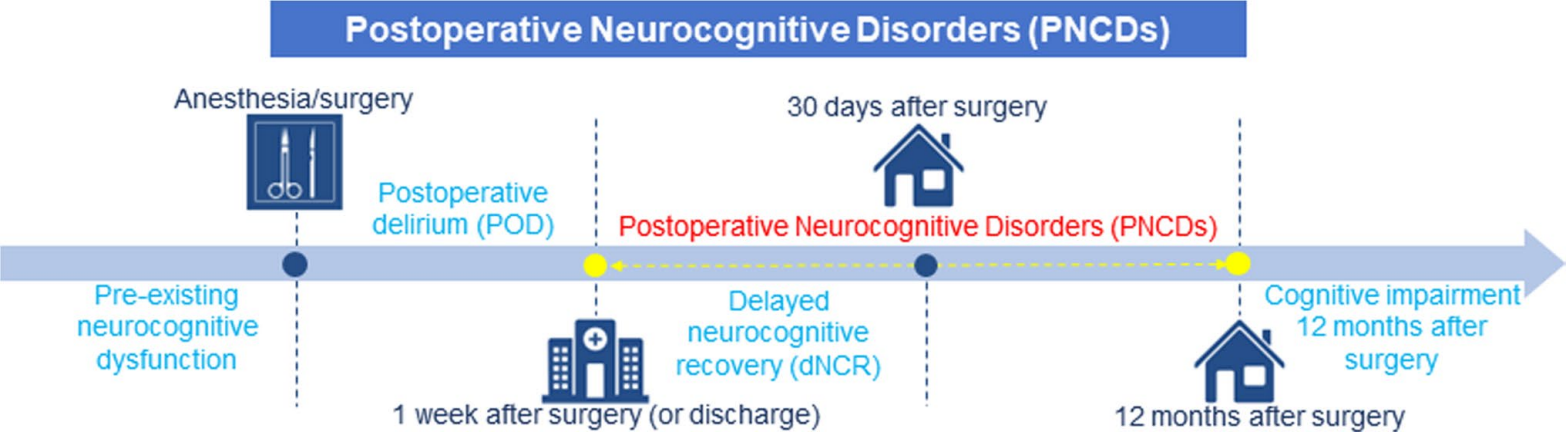
Nomenclature of PNCDs. PND including pre-existing neurocognitive dysfunction, POD (occurred within 1 week after surgery or before discharge), dNCR (cognitive decline within 7–30 days after surgery), PNCDs (mild and severe cognitive decline existed from 7 days to 12 months after surgery), and first diagnosed cognitive impairment 12 months after surgery

Postoperative brain dysfunction is a common complication in elderly patients after anesthesia and surgery, significantly impacting their daily functionality and long-term quality of life. In 2018, consensus recommendations from a multidisciplinary expert panel suggested using the term “perioperative neurocognitive disorders (PND)” to describe changes in cognitive function in patients during the perioperative period. These cognitive disorders include pre-existing cognitive decline, postoperative delirium (POD), delayed neurocognitive recovery (dNCR), postoperative neurocognitive disorders (PNCDs), and cognitive decline occurring 12 months after surgery [31, 32] (Fig. 1). PNCDs specifically refer to cognitive impairments diagnosed within the 7-day post-surgery or within 12 months after hospital discharge, clinically manifested by reduced cognitive ability, attention, memory, and personality changes. PNCDs not only severely impact the postoperative quality of life of patients but also increase mortality, hospitalization duration, complications, and the burden on social healthcare and families. However, existing findings exhibit contradictions or controversies. Despite various pathological factors being implicated in the onset of PNCDs, the pathophysiological basis and specific mechanisms underlying these disorders remain unclear, and effective prevention and treatment methods are yet to be established.

### Risk Factors for PNCDs

The increasing incidence of PNCDs is correlated to the growing number of surgeries and elderly patients. Factors influencing the occurrence of PNCDs include preoperative comorbidities, intraoperative conditions, effects of anesthesia and surgery, and postoperative situations [33]. Among these, age is currently confirmed as one of the most closely associated independent factors with the onset of PNCDs [34]. Elderly patients may exhibit age-related neurodegenerative changes preoperatively, along with comorbidities such as diabetes, hypertension, and other chronic diseases affecting multiple systems. During the perioperative period, patients may experience conditions such as hypotension and hypoxia, which can consequently trigger the onset of PNCDs [33]. The risk of PNCDs increases by 2.6 times for each increased score, according to the preoperative American Society of Anesthesiologists (ASA) grade [35–37]. Patients with lower levels of education, preoperative fasting-induced metabolic disruptions, or preoperative bleeding and transfusions can also increase the risk of PNCDs [38].

During surgery, various potential factors can also induce the development of PNCDs, including but not limited to anesthesia-related factors, intraoperative blood pressure and temperature fluctuations, and surgical trauma [39, 40]. Implementing depth of anesthesia monitoring in elderly patients during surgery would be more beneficial in preventing stress responses caused by inadequate anesthesia, thereby reducing peripheral inflammation and the occurrence of short-term postoperative cognitive function changes [41]. The depth of general anesthesia, type of surgery, and choice of anesthetic agents can all have varying effects [42]. For instance, sevoflurane may have neuroprotective properties, improving early postoperative cognitive dysfunction [43], and propofol can reduce the incidence of delayed neurocognitive recovery [44]. Importantly, recent views increasingly correlated the occurrence of PNCDs with surgical trauma-induced stress and inflammatory storms. Surgical operations provoke strong stress responses, leading to a sudden increase in glucocorticoid levels and the release of pro-inflammatory factors. Concurrently, dysfunction of the blood-brain barrier allows peripheral pro-inflammatory factors to enter the central nervous system, causing neuroinflammation and activating the immune system in the brain, thereby reducing the expression of brain-derived neurotrophic factor (BDNF) in the hippocampus and inducing PNCDs [45, 46]. Different types of surgeries and degrees of surgical trauma may be associated with varying impacts on hippocampal neuroinflammatory markers and postoperative cognitive functions [47], suggesting that perioperative neuroinflammation due to surgical trauma might be more closely related to cognitive changes than various anesthetic factors.

Improper postoperative management, including postoperative infection, postoperative delirium, and painful irritation, may increase the incidence of PNCDs [48]. Persistent inflammation and pain after surgery are both important risk factors for the progression of PNCDs. Studies have found that elevated serum levels of inflammatory factors such as IL-6 and TNF-α postoperatively can serve as biomarkers for predicting postoperative delirium [49, 50]. Non-steroidal anti-inflammatory drugs (NSAIDs) are often used as adjuvant analgesics in perioperative analgesia, which can provide effective analgesia and reduce the inflammatory response caused by surgery. NSAIDs such as parisibna, acetaminophen, ibuprofen, and flurbiprofen have been proven to reduce the incidence and severity of PNCDs by reducing postoperative pain and inflammatory response [51–53].

With advancing research into the realm of gut microbiota, it was implicated that gut microbiota may also participate in the development of PNCDs through various regulations [10, 54]. Research has found that disturbing the homeostasis of gut microbiota in mice by administering antibiotics before surgery increases the incidence of PNCDs. Conversely, using the beneficial metabolite sodium butyrate (NaB) can improve gut dysbiosis in mice and reduce the probability of PNCD occurrence [10].

### The Mechanism of PNCDs

With the in-depth study of the mechanism of PNCDs, it has been proven that inflammation, oxidative stress, microglia activation, mitochondrial dysfunction, and increased blood-brain barrier (BBB) permeability are involved in the pathogenesis of PNCDs. Currently, it is believed that the occurrence of PNCDs is mainly associated with neuroinflammation induced by perioperative stress [55]. Neuroinflammation in PNCDs mainly originates from the infiltration of peripheral pro-inflammatory cytokines. Stress responses induced by anesthesia and surgery lead to the release of damage-associated molecular patterns (DAMPs), with high-mobility group box 1 (HMGB1) being particularly significant. HMGB1 activates NF-κB by binding to toll-like receptors on immune cells, triggering systemic pro-inflammatory cytokine release [56]. These cytokines enhance local prostaglandin synthesis by upregulating cyclooxygenase-2 (COX-2) and matrix metalloproteinases (MMPs), undermining the permeability of the blood-brain barrier [57]. Subsequently, pro-inflammatory factors penetrate the compromised blood-brain barrier and promote the polarization of microglial cells toward the M1 phenotype (pro-inflammatory phenotype), releasing pro-inflammatory factors, thus amplifying central nervous system inflammation [50]. Additionally, the release of reactive oxygen species triggers the HMGB1 response, further exacerbating neuroinflammation and resulting in sustained neurophysiological dysregulation in critical cognitive-related regions such as the hippocampus. This ultimately leads to a decline in learning and memory abilities and cognitive dysfunction [58–60]. The immune communication between the periphery and the central nervous system is mediated by various molecular pathways involving multiple cell types and cannot be attributed to a single pathway regulation. Generally, stress-induced immune dysregulation leads to amplification of peripheral signaling, which acts on the central nervous system, further influencing immune responses within the brain and inducing cognitive dysfunction.

Neuroinflammation in PNCDs may also relate to disruptions in the cholinergic anti-inflammatory pathway (CAP) [61]. The CAP is a regulatory mechanism between the central nervous system and the immune system, which plays a crucial role in cognitive function by inhibiting inflammation in the nervous system. Upon sensing peripheral pro-inflammatory cytokines, the vagus nerve signals to the celiac ganglion, activating β2-adrenergic receptors on T lymphocytes, upregulating acetyltransferase transcription, and promoting the synthesis of acetylcholine (ACh). ACh activates macrophages expressing α-7 nicotinic ACh receptors (α7nAChRs) in circulation, leading to the inactivation of NF-kB and ultimately reducing pro-inflammatory cytokine release [57]. Results from a prospective observational cohort study indicated that increased perioperative peripheral acetylcholinesterase (AChE) activity and decreased butyrylcholinesterase (BuChE) activity would increase the occurrence of POD [62]. Research by Kalb et al. showed that AChE inhibitors like physostigmine and neostigmine significantly reduce the expression of IL-1 and TNF-α protein, mitigating post-surgery pro-inflammatory responses in the cortex and hippocampus and inhibiting neuroinflammation [63]. Studies by Saito et al. also indicated that epigenetic regulation of cholinergic genes is involved in the development of postoperative delirium [64]. While the relationship between the cholinergic system and neuroinflammation has been established, its correlation to the onset of PNCDs and associated clinical significance remains unclear. Pathological processes like mitochondrial dysfunction [65], epigenetic spectrum changes [66, 67], blood-brain barrier damage [59], and oxidative stress [68] are also involved in the onset of PNCDs.

Recent findings suggest that disruptions in the gut microbiota and gut homeostasis can influence the progression of PNCDs. Modulation of the gut microbiota and/or gut microecology and restoration of the damaged gut barrier can mitigate the progression of PNCDs. Studies have shown that preoperative treatment with cefazolin in mice undergoing laparotomy improves anesthesia and surgery-induced behavioral changes while reducing IL-6 and IL-1β expression in the hippocampus, cortex, and colon 24 h postoperatively [69]. By using aged mice to establish a model of PNCDs following tibial fracture internal fixation surgery, researchers have confirmed alterations in the abundance of 37 bacterial genera in the mice. Further investigation has determined that the cognitive impairment induced by anesthesia/surgery is mediated by dysbiosis of the gut microbiota, and pretreatment with antibiotics can prevent learning and memory impairment [13]. These findings suggest that the gut microbiota likely plays a role in the pathogenesis of postoperative neurocognitive disorders, with complex mechanisms of action during disease progression. Therefore, clarifying the contribution of the gut microbiota to PNCDs is vital, as it may help to identify potential biomarkers and provide potential therapeutic approaches for PNCDs.

## Gut Microbiota Dysbiosis and PNCDs

It is speculated that potential communicational pathways exist between the gut microbiome and the brain, for instance, the gut-brain axis, which serves as a bidirectional signaling mechanism between the two factors. The transition of gut microbiota structure from a healthy phenotype to a pathogenic phenotype is a major mechanism through which intestinal bacteria induce the onset of neurological and psychiatric disorders via the gut-brain axis. Gut microbiota interacts with the brain primarily through the intestinal barrier and BBB, immune system, hypothalamic-pituitary-adrenal (HPA) axis, metabolic system, and vagus nerve [70]. From the bottom up, intestinal bacteria can affect central nervous system function by synthesizing and secreting neurotransmitters, neurotrophic factors, or metabolites. In addition, the vagus nerve distributes a large number of receptors for intestinal regulatory peptides and intestinal metabolites, which represent yet another main way for intestinal bacteria to affect the central nervous system. The aberrant composition of the gut microbiota can also disrupt the integrity of the intestinal barrier, activate the immune system, and trigger systemic inflammation. This, in turn, can compromise the blood-brain barrier, leading to abnormal activation and increased numbers of microglial cells, ultimately resulting in neuroinflammation and neuronal damage. From the top down, the brain can influence the sensory, motor, and secretory functions of the gut, transmitting information to the gastrointestinal tract through complex neurohumoral pathways within the gut-brain axis, such as the HPA axis and the autonomic nervous system.

**Fig. 2:**
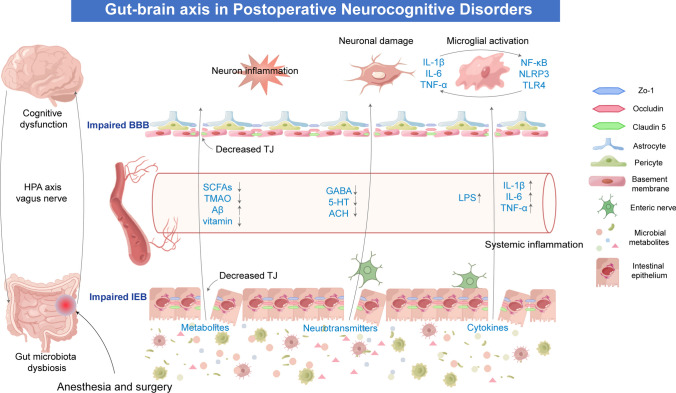
The mechanism of the role of gut-brain axis in PNCDs. The gut microbiota plays a pivotal role in modulating communication between the gut and the brain, operating via immune responses, metabolites, and neurological and endocrine pathways. Disruption of the gut microbiota following anesthesia and surgery leads to the dysregulation of intestinal immune activity, resulting in the production of inflammatory cytokines. This initiates systemic inflammation, subsequently compromising both the intestinal barrier and the blood-brain barrier, allowing the infiltration of inflammatory agents into the central nervous system. Consequently, this cascade contributes to an escalation in aberrant microglial cell activation. These cytokines additionally stimulate the development of neuroinflammation and further activate the HPA axis. Disruptions in the gut microbiota can prompt alterations in the levels and functions of metabolites. This may involve a reduction in beneficial metabolites such as short-chain fatty acids (SCFAs) and vitamins, accompanied by an elevation in detrimental metabolites like trimethylamine N-oxide (TMAO) and lipopolysaccharides (LPS). These changes subsequently influence the onset of postoperative neurocognitive disorders (PNCDs). This also has the potential to reduce levels of gamma-aminobutyric acid (GABA), serotonin (5-HT), and acetylcholine (ACH). Aberrant metabolites, neurotransmitters, and inflammatory mediators are conveyed to the central nervous system through the gut-brain axis, inducing neuroinflammation and neuronal damage. Multiple pathways collaboratively contribute to the manifestation of neurocognitive dysfunction

The composition of the gut microbiota can change dynamically in response to external factors such as aging, environmental changes, alterations in daily dietary intake, and medication stimuli. Numerous results from animal model-based experiments indicate that anesthesia and surgery can induce changes in the composition and structure of the gut microbiota in animals. The altered gut microbiota can negatively impact cognitive function through various pathways [71]. Furthermore, by applying antibiotics to deplete the gut microbiota in mice and creating a model resembling germ-free mice, the influence of postoperative changes induced by gut microbiota could be eliminated. This procedure can improve memory capacity in aged mice following surgery. However, clinical evidence is currently lacking, with randomized controlled clinical trials to explore the intervention of probiotics in perioperative gut dysbiosis ongoing. Given the crucial role of the gut microbiota in regulating cognitive impairment, some scholars have further speculated that targeting probiotics may reduce the incidence of PNCDs in elderly patients following surgery [72]. The bidirectional signaling of the gut-brain axis may be a critical factor in how the gut microbiota influences the occurrence of PNCDs (Fig. 2).

### Migration of Inflammatory Factors from Gut to Brain

The maturation of gut-associated lymphoid tissue plays a decisive role in developing the immune barrier system. Approximately 70% of the immune cells are located in the gut, and the interaction between gut microbiota and the host is crucial for the development and maintenance of the immune system. The innate immune system of the gut is tolerant to the normally distributed symbiotic microbiota. However, when the stability of the gut microbiota is disrupted, it stimulates local or systemic immune responses in the host. Gut dysbiosis, characterized by an increased proportion of Gram-negative bacteria, leads to the synthesis of lipopolysaccharides (LPS) with potent pro-inflammatory effects. LPS can cross the compromised gut barrier and blood-brain barrier, activating microglia and exacerbating central neuroinflammation [73]. Activation of the gut immune system may be a critical factor in this process. Postoperative changes in intestinal bacterial counts in patients undergoing gastrointestinal surgery often lead to an increased proportion of potential pathogens and a decrease in beneficial bacteria like Lactobacillus and Bifidobacterium [16]. When there is dysbiosis in the gut microbiota, bacterial-associated molecular patterns produced by pathogenic bacteria bind to pattern recognition receptors on host cells, producing pro-inflammatory cytokines and increasing entry into the bloodstream. This triggers peripheral inflammation, exacerbating neuroinflammatory responses. This process represents the migration of inflammatory factors from the periphery to the central nervous system. Studies have shown that an increased abundance of the probiotic Akkermansia can enhance metabolic levels in patients with obesity and type II diabetes, protect the gut barrier, and reduce inflammation by inhibiting the LPS-TLR4/NF-κB signaling pathway [74]. TLR4-mediated enterogenic inflammation and gut dysfunction may exacerbate neurodegeneration and central neuroinflammation in Parkinson’s disease patients. These patients show a reduced abundance of short-chain fatty acids (SCFA)-producing bacteria in the gut, colonic mucosal inflammation, and increased expression of bacterial endotoxin-specific ligand TLR4 and CD3 + T cells [27]. Studies have found that tibial fractures increase the expression of peripheral interleukin 17 A (IL17A) in the serum and hippocampus of elderly mice, leading to central inflammation and neurocognitive disorders. Preoperative administration of antibiotics to deplete gut microbiota significantly reduces Th17 cell numbers and downregulates IL17, IL17R, and inflammatory cytokine levels, ultimately improving memory function [54]. Thus, the Th17 cells and IL-17 may be essential bridges through which gut microbiota influence neuroinflammation.

Numerous central nervous system diseases, including PNCDs, are correlated to the onset of perioperative stress-induced central neuroinflammation [50], with nucleotide-binding oligomerization domain-like receptor protein 3, NLRP3 (NLRP3) inflammasome-mediated neuroinflammation, and microglial activation being key factors in exacerbating these diseases [75]. In sleep deprivation-induced cognitive impairment, chronic sleep deprivation induces gut dysbiosis in mice, activating NLRP3 inflammasomes in the colon and brain. This phenomenon disrupts the gut/brain barrier, impairing cognitive function in mice. Downregulating intestinal NLRP3 expression can protect the gut barrier, reduce peripheral inflammatory cytokine levels, downregulate brain NLRP3 expression, and improve cognitive function [76]. The increased expression of inflammasomes due to gut microbiota dysbiosis may serve as the initiating condition for upregulating downstream pro-inflammatory cytokines and cytotoxic mediators. NLRP3 inflammasomes, in particular, serve as vital regulatory factors for neuroinflammation influenced by the gut-brain axis via the gut microbiota [77].

The dysregulated communication between the peripheral and central immune systems induced (triggered by the gut microbiota dysbiosis) can increase levels of pro-inflammatory cytokines in the hippocampus, thus disrupting the balance between host pro-inflammatory and anti-inflammatory mechanisms. This imbalance, exacerbated by functional disruption of the gut-brain axis, worsens cognitive impairment, playing a significant role in PNCDs triggered by gut microbiota dysbiosis. Therefore, reshaping dysbiotic gut microbiota may represent a potential strategy for treating inflammation-related neurological disorders (Fig. 2).

### Microbial Metabolites

The gut microbiota can provide the host with biological enzymes and metabolic pathways that the host itself does not possess, facilitating the production of short-chain fatty acids, vitamins, neurotransmitters, and metabolic products such as LPS, which are transferred to the circulatory system. Consequently, they regulate the microenvironment of the brain and brain function. Therefore, dysbiosis of the gut microbiota can lead to abnormalities in various metabolic product levels [78].

Beneficial bacteria in the gut, such as *Bifidobacteria*, *Lactobacilli*, and *Bacteroides*, can ferment dietary fiber to produce SCFAs, including butyrate, acetate, and propionate. SCFAs play crucial roles in the physiology and pathology of the host. They are the main energy source for intestinal epithelial cells and could facilitate the differentiation of regulatory T cells, which are involved in immune modulation. SCFAs may affect gut-brain communication and brain function through immune, endocrine, vagal nerve, and other bodily fluid pathways through direct or indirect modulations. Research shows that SCFAs can directly activate vagal afferent nerves through free fatty acid receptors, thereby sending signals to the brain to regulate eating behavior and other brain functions [79]. Recent studies have also found that SCFAs can enhance the expression of tight junction (TJ) proteins between blood-brain barrier endothelial cells (ECs), protecting BBB integrity [80]. They can also enter the central nervous system and modulate microglial activation to exert anti-inflammatory effects. Feng et al. discovered that SCFAs could inhibit LPS-induced activation of NLRP3 inflammasomes and autophagy, protecting the gut barrier from LPS damage and stimulating its formation [81]. However, contrasting evidence suggests that SCFAs can increase the expression of NLRP3 activation and inflammatory cytokines (IL-18, IL-6, and TNF-α) in the gut, leading to neuroinflammation upon reaching the central system and increasing the levels of inflammatory cytokines in the cerebrospinal fluid (CSF) of AD mice, exacerbating their cognitive impairments. In a co-culture system of double-negative T cells (CD3 + CD4 − CD8−, DNTs) and intestinal macrophages, SCFAs can promote the formation of DNTs, inducing the activation of NLRP3 inflammasomes and the release of inflammatory cytokines within macrophages through the Fas/FasL-TNF-α signaling pathway. This could be a new mechanism of neuroinflammation in Alzheimer’s disease and potentially also in pathological neurological disorders (PNDs) with similar pathological bases [82].

Trimethylamine N-oxide (TMAO) is a unique metabolite produced by gut microbiota, typically excreted by the kidneys under normal physiological conditions [83]. When there is dysbiosis of the gut microbiota or impaired kidney function, circulating levels of TMAO increase, and the TMAO can induce oxidative stress and inflammation in peripheral tissues and rapidly penetrate the blood-brain barrier to induce neuroinflammation. In the gut fecal samples from elderly individuals, levels of trimethylamine (TMA) and its precursors, such as propionate and choline, are elevated, potentially increasing the expression of pro-inflammatory cytokines IL-8 and IL-21 [84]. Studies suggest that TMAO could be a potential biomarker for aging brains and neurocognitive disorders, with higher expression levels in the circulation of elderly individuals, Alzheimer’s disease patients, and those with cognitive impairments related to non-infectious diseases [85]. In mice, plasma and brain tissue concentrations of TMAO increase with aging, which can promote the expression of pro-inflammatory cytokines and activation of astrocytes in the brain, leading to neuroinflammation and cognitive dysfunction [86]. In a rat model of postoperative cognitive dysfunction (POCD, treatment with TMAO after laparotomy significantly reduced the expression of the antioxidant enzyme methionine sulfoxide reductase A (MsrA), further increased neuroinflammation mediated by astrocytes, and enhanced the formation of reactive oxygen species (ROS) in hippocampal tissue. This exacerbated the susceptibility to oxidative stress induced by surgery and worsened cognitive decline and neuroinflammation in aged rats [87]. These findings also suggest that reducing circulating TMAO levels during the perioperative period may be a novel strategy for preventing POCD, and this goal could potentially be achieved through interventions targeting the gut microbiota.

The gut microbiota can also utilize dietary-derived amino acids to synthesize other metabolites. For example, lactic acid bacteria, such as *Lactobacillus* and *Bifidobacterium*, participate in the synthesis of gamma-aminobutyric acid (GABA) using glutamate. GABA synthesized by gut microbiota can stimulate enterochromaffin cells in the gut to secrete serotonin (5-HT), influencing the production of brain-derived neurotrophic factors and dopamine. Additionally, gut microbiota can directly affect the metabolism of tryptophan, thereby modulating 5-HT signaling. Studies have indicated that gut microbiota might play a significant role in depression through the tryptophan-kynurenine (trp-kyn) metabolic pathway. Approximately 95% of the serotonin is synthesized by enterochromaffin cells in the gut, and its regulation of vagal nerve input and intestinal inflammatory responses can affect signaling along the gut-brain axis. Indole derivatives, which are tryptophan metabolites, can beneficially impact neurocognitive function by maintaining intestinal environment stability and mitigating LPS-induced neuroinflammation [88]. Anesthesia and surgery can disrupt the gut microbiota, impairing the stability of 5-HT synthesis and metabolism involving enteroendocrine cells and gut microbiota. This disruption can lead to fluctuations in the 5-HT levels, subsequently affecting postoperative cognitive function. Bacteria like Lactobacilli and bacilli can also metabolize to produce acetylcholine (ACh), altering levels of neurotransmitters in the body. In Alzheimer’s disease patients, impaired expression of ACh can lead to cognitive impairments and inflammation in the central and peripheral nervous systems, further inducing the release of pro-inflammatory cytokines [89].

Additionally, there are other metabolic products related to gut microbiota, such as LPS from the cell walls of Gram-negative bacteria. LPS is a potent activator of TLR-4 on intestinal epithelial cells, capable of diminishing the intestinal epithelial barrier, thus increasing its permeability. This effect will lead to immune responses and activation of the HPA axis. LPS can also enter the brain through circulation and activate microglia, causing neuroinflammation and impairing cognitive functions [30]. PNCDs share similar neuropathological mechanisms with Alzheimer’s disease, where anesthesia and surgery can elevate the expression of beta-amyloid (Aβ), ultimately leading to cognitive impairments [90]. Gut microbiota-synthesized or food-derived B vitamins, such as pyridoxine (vitamin B6), folate (vitamin B9), and cobalamin (vitamin B12), have been shown to improve cognitive abilities in patients with mild cognitive impairment (MCI) [91]. Bacteria like *Escherichia coli*, *Bacillus subtilis*, and *Salmonella *spp. can produce a significant amount of amyloid proteins, which can enter the bloodstream through a damaged intestinal barrier. Within the gut, the increase in the abundance of *E. coli *and *Shigella* spp., along with gut microbiota dysbiosis, can lead to an abnormal accumulation of gut-derived Aβ proteins, further exacerbating neurocognitive impairments.

The evidence presented highlights the close correlation between gut microbiota metabolites and cognitive impairments in various central nervous system diseases. Postoperative dysbiosis of gut microbiota could disrupt intestinal homeostasis and metabolic balance, altering the levels and functions of metabolites, which in turn could influence the occurrence of PNCDs. Animal model-based studies provide direct evidence for the impact of microbiota metabolites on central nervous system (CNS) function. However, clinical research in humans is limited, and the methodological constraints have led to inconsistent findings in most experiments. Future research necessitates more clinical studies to confirm these conclusions. More clinical research is needed to confirm these findings. Targeting gut microbiota metabolites to improve neurocognitive functions presents a new potential strategy for preventing and treating PNCDs (Fig. 2).

### Nervous System and the Neuro-Neuroendocrine Axis

The brain communicates with the gut through various neuroendocrine pathways, including the vagus nerve, the enteric nervous system (ENS), and the HPA axis. Signals from the gut can regulate brain functions by influencing immune responses and the endocrine system. Patients with disorders in the central nervous system were identified to exhibit gastrointestinal symptoms as complications.

The vagus nerve, a major component of the parasympathetic nervous system, is a key neural pathway between the gut microbiota and the brain. The afferent fibers of the vagus nerve are distributed throughout the layers of the gastrointestinal tract. Although they do not directly contact the gut microbiota or substances within the gut, the ENS, upon stimulation by gut-derived signals, transmits neurophysiological signals about the internal physiological state of visceral organs to the central nervous system via the afferent vagus nerve [92]. This conversation includes sensing microbiota-related neurotransmitters, metabolites, and hormones. Based on recent findings, altering the transmission of vagal nerve signals by modulating the gut microbiota or through surgical procedures such as vagotomy can induce anxiety- and depression-like behaviors [93]. Conversely, the improvement of anxiety and depression symptoms can be achieved through vagus nerve stimulation (VNS) or appropriate modulation of vagal nerve signaling [94]. However, when gut microbiota loses its homeostasis, the vagus nerve can influence brain functions indirectly by modulating the endocrine pathway, affecting the secretion of corticotropin-releasing factor (CRF). The CRF, in turn, increases the permeability of the intestinal barrier through TNF-α and proteases from mast cells. Regarding the impact of the vagus nerve on the central nervous system, there are currently conflicting viewpoints. Some researchers propose that the gut microbiota enhance the aggregation of alpha-synuclein (α-syn) and other similar proteins, potentially facilitating the spread of pathogens to the central nervous system through the vagus nerve. Conversely, others refute the pathology of α-synuclein in Parkinson’s disease, suggesting that it does not spread to the central nervous system through the vagus nerve [95]. Further exploration is needed to elucidate the specific mechanisms by which the vagus nerve influences neurodegenerative diseases.

The HPA axis is a crucial component of the neuroendocrine system and is involved in various stress responses. Recent research has discovered that gut microbiota can activate the HPA axis through multiple mechanisms, such as microbial antigens, cytokines, and prostaglandins, thereby affecting brain function. The central nervous system can also regulate the permeability of the gut barrier, immune cells, and the composition of symbiotic gut microbiota through cortisol released by the HPA axis [96]. Experiments with germ-free mice have shown that stability in the gut environment aids in the social activities of mice. Specific intestinal bacteria can inhibit the activation of the HPA axis, and the typical stress responses and social behaviors can be modulated by inhibiting the corticosterone levels mediated by the HPA axis [97]. Dysbiosis of gut microbiota can cause pro-inflammatory cytokines like IL-1β, IL-6, and TNF-α to activate the HPA axis via the blood-brain barrier. This feedback mechanism exacerbates intestinal barrier dysfunction and spreads entheogenic inflammation, further affecting the occurrence of PNCDs. Although research in this area is limited, the role of the HPA axis in the influence of gut microbiota on PNCDs should not be overlooked (Fig. 2).

### The Pathogenetic Role of Intestinal Barrier Dysfunction

The permeability of the intestinal barrier, governed by its integrity, is influenced by alterations in gut microbiota composition, impacting the permeability of both the intestinal epithelial barrier and mucosal layers. Thinning of the mucosal barrier caused by aging or disruption of intestinal barrier integrity can lead to the migration of microbiota and metabolites into the systemic circulation or other tissues, resulting in inflammation and immune activation [98]. Certain products of gut microbiota, such as bacterial products like endotoxins, can disrupt the integrity of intestinal epithelial cells. Conversely, probiotics such as *Lactobacillus plantarum* and *Bifidobacterium* can upregulate the expression of tight junction proteins, maintaining intestinal barrier integrity to protect the host from the harmful effects of substances within the gut. The intestinal barrier plays a critical role in controlling the entry of specific bacterial products, including SCFAs, vitamins, and neurotransmitters, into the bloodstream and, eventually, the brain. The impaired function of the intestinal barrier is a pivotal event in gut-brain axis dysfunction. A state of dysbiosis or elevated gut permeability typically signifies an inflammatory intestinal milieu, which can aggravate impairments in brain function [99]. Under normal conditions, LPS, key constituents of Gram-negative bacterial cell walls, are contained by a healthy intestinal barrier. However, in Alzheimer’s disease patients, plasma LPS concentrations are markedly increased compared to those in healthy individuals [100]. In the context of PNCDs, gut microbiota alterations influence intestinal barrier functionality, contributing to increased permeability, commonly referred to as "leaky gut," and a consequent role in neuroinflammation [6]. A cohort study indicated that post-surgical dysbiosis and compromised intestinal barrier in elderly orthopedic patients may underlie postoperative cognitive decline, noting a post-surgery decrease in SCFA-producing bacteria and an increase in Gram-negative bacteria, potentially linked to surgery-related perioperative metabolic stress and inflammatory responses [100]. The infiltration of detrimental agents into the bloodstream via a compromised intestinal barrier may also undermine the BBB’s integrity, further provoking neuroinflammatory processes. Currently, there is limited data regarding the involvement of dysfunctional intestinal barriers in the pathogenesis of PNCDs, highlighting the urgent need for the assessment of gut barrier-derived biomarkers for central nervous system diseases. Beyond modulating gut microbiota, targeting the intestinal barrier could serve as a therapeutic approach for central nervous system disorders (Fig. 2).

### The Contribution of Blood-Brain Barrier Disruption

The BBB shares a similar cellular composition with the intestinal epithelial barrier and serves as a unique physical barrier between the central nervous system and the peripheral circulation. It is comprised of brain microvascular endothelial cells (BMECs), astrocytes, and neurons, among other components. The BBB effectively prevents the entry of peripheral inflammatory factors, harmful metabolites, and immune cells into the brain. Among these, BMECs are connected by TJ proteins. Dysfunction of the BBB is typically associated with disruption or excessive permeability of the tight junctions between endothelial cells. Compromised integrity allows peripheral inflammatory molecules to influence permeability through the upregulation of enzymes like cyclooxygenase-2 and matrix metalloproteinases of this barrier. This condition enables pro-inflammatory factors to enter the central nervous system, activating microglia and triggering an inflammatory response [101]. Notably, the correlation between neurodegenerative diseases and dysfunction of this barrier has been established [102]. Similarly, a key pathological mechanism in POD among elderly patients involves increased permeability of this barrier [103]. Post-surgery changes in the gut microbiota can lead to the release of pro-inflammatory cytokines, such as IL-1β, IL-6, and TNF-α, activating inflammatory signaling pathways and contributing to damage to this barrier. Pro-inflammatory cytokines and harmful substances from the gut entering the brain through a compromised barrier can initiate immune and inflammatory responses, causing neuronal and synaptic damage. Production of short-chain fatty acids by gut microbiota from carbohydrate breakdown, particularly sodium butyrate, can reduce the permeability of this barrier. Sodium butyrate maintains the expression of tight junction proteins between endothelial cells of the barrier, improving its integrity. Research has revealed a decrease in gut probiotics leads to a reduction in NaB production, which may enhance BBB permeability. Conversely, *Lactobacillus* and its production of NaB can increase the expression of tight junction proteins between ECs, thereby inhibiting BBB permeability and improving postoperative cognitive function in elderly mice [11]. Therefore, preventing postoperative dysbiosis of gut microbiota is an effective approach to prevent BBB impairment and consequent PNCDs (Fig. 2).

## Gut Microbiota Modulation and Its Application in the Prevention and Treatment of PNCDs

With the adoption of postoperative rehabilitation therapy, an increasing number of practical measures are being implemented clinically to reduce perioperative stress responses and complications. The prevention of PNCDs relies primarily on preventive strategies for perioperative risk factors [48]. Enhanced postoperative management of patients, including early initiation of postoperative pain management and cognitive training, as well as early identification of postoperative complications, may help reduce the risk of PNCDs. Evidence from existing research has confirmed that disturbances and destabilization of gut microbiota would affect the progression of PNCDs. Studies are currently underway to investigate the modulation of gut microbiota as a potential therapeutic approach to restore gut microbiota homeostasis, repair compromised intestinal barriers, and ultimately alleviate PNCDs, also known as target gut microbiota for the treatment of PNCDs, including probiotics and prebiotics, fecal microbiota transplantation, and dietary regulation that can be readily accepted by clinical patients [104]. To understand the current status of the application of gut microbiota modulation in the prevention and treatment of PNCDs, our present review summarizes the clinical or preclinical findings related to gut microbiota modulation in PNCDs, listed in Table 1.

**Table 1:**
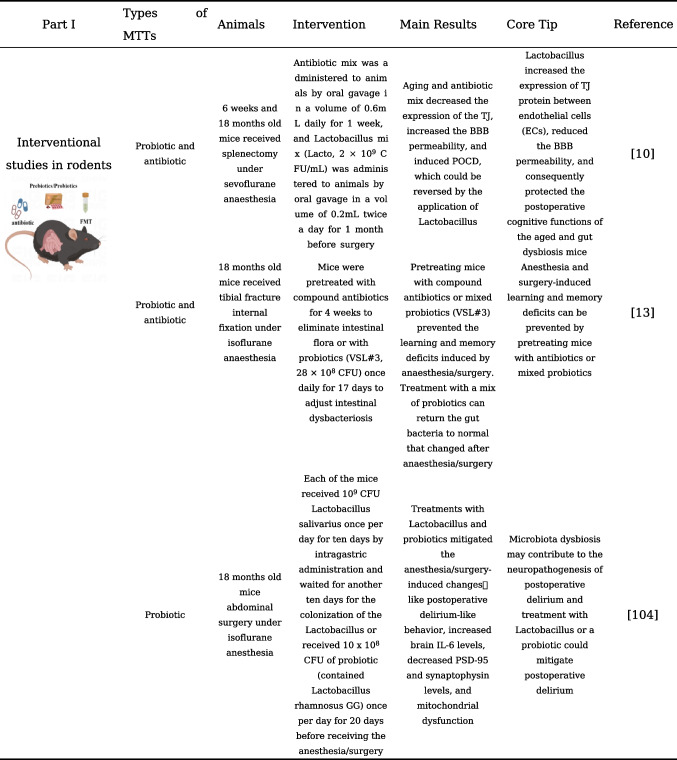

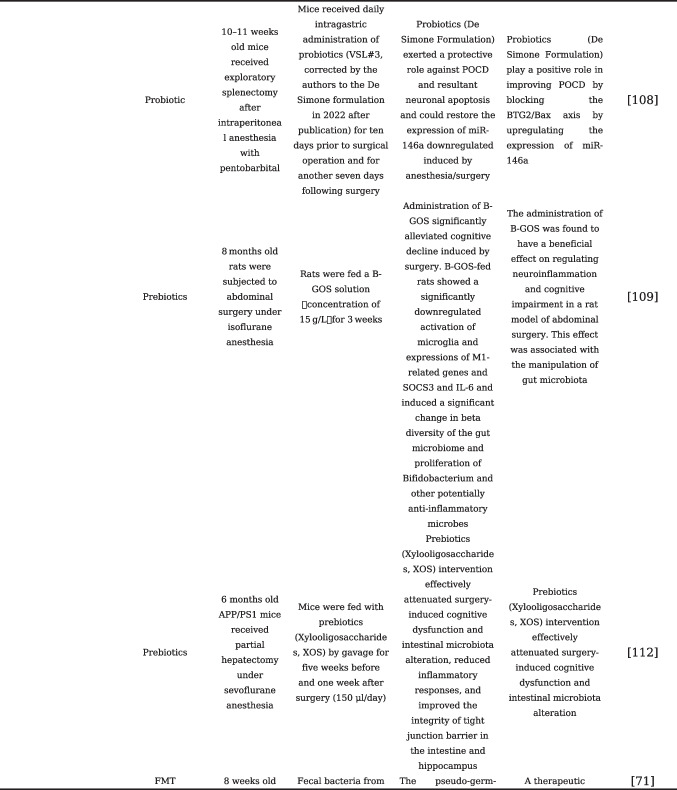

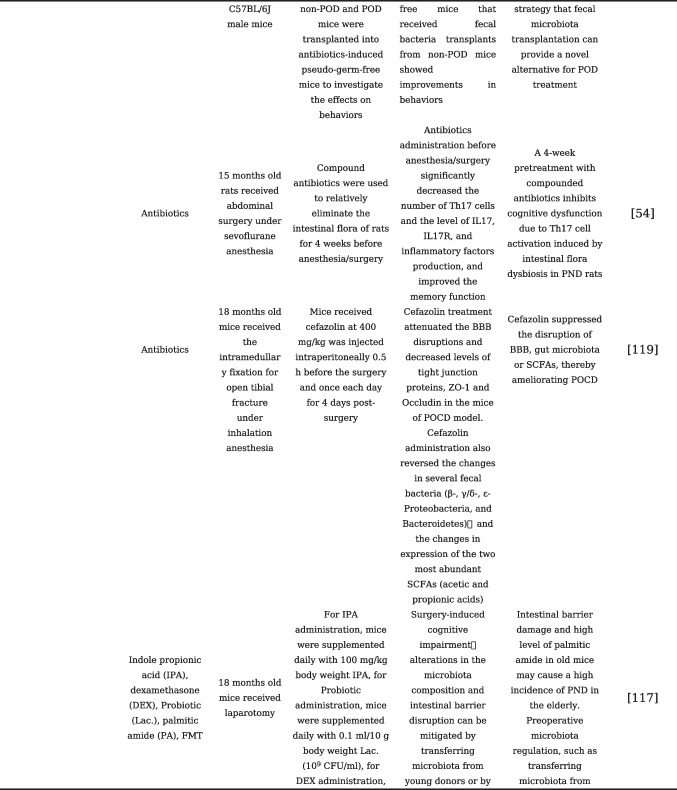

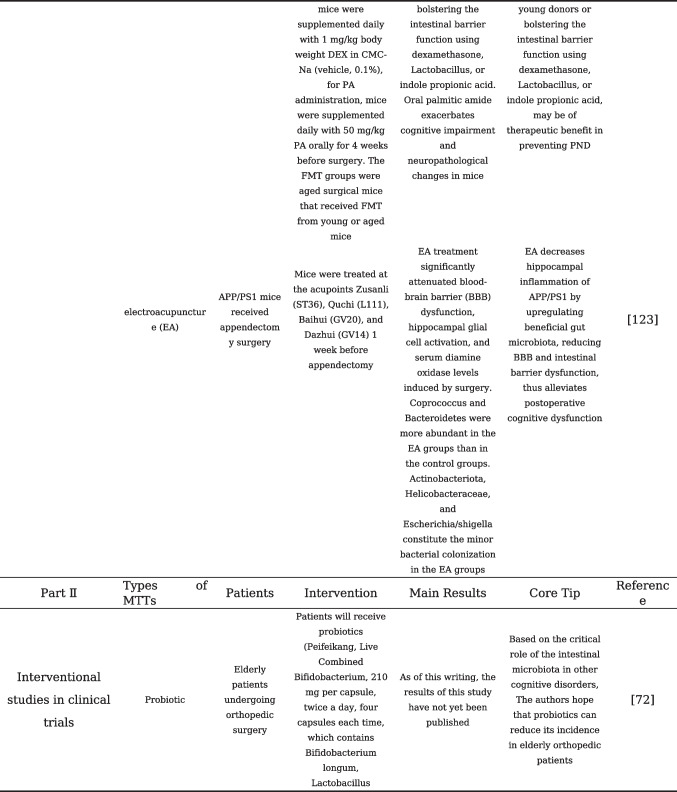

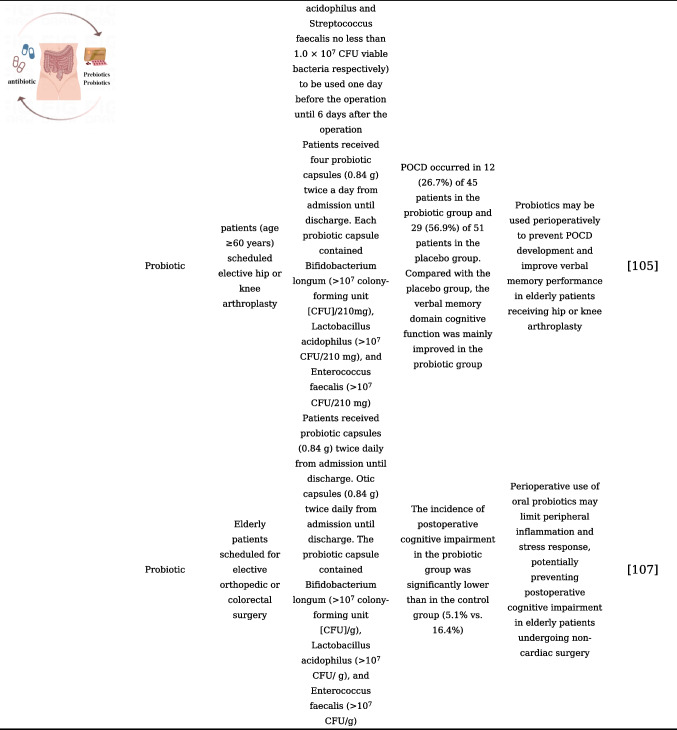
Gut microbiota modulation and its application in the prevention and treatment of PNCDs

### Probiotics and Prebiotics

Probiotics are beneficial microorganisms involved in maintaining host health. *Lactobacillus* and *Bifidobacterium* are the most widely used probiotics in clinical practice. Probiotics can exert anti-inflammatory effects by modulating the NF-κB signaling pathway and NLRP3 inflammasome, thereby impacting intestinal immune activation. They can also reduce intestinal permeability. Studies have found that probiotics can prevent PNCDs and improve verbal memory in elderly patients undergoing hip or knee replacement surgery [105]. Using adult and elderly mice to construct an abdominal surgery model, researchers found that elderly mice exhibited more severe delirium-like behavior postoperatively, along with significant changes in gut microbiota characterized by the decreased relative abundance of *Lactobacilli*. Additionally, their brain tissue showed significantly increased levels of IL-6 and lower levels of postsynaptic density protein 95 (PSD-95) and synaptophysin. Preoperative treatment with probiotics and *Lactobacilli *significantly alleviated postoperative delirium in mice [106]. In a prospective cohort study, researchers administered probiotics or a placebo to 120 elderly patients undergoing orthopedic or colorectal surgery. The results showed that patients receiving probiotic treatment had a significantly lower incidence of postoperative cognitive impairment. Additionally, postoperative plasma levels of IL-6 and cortisol were lower in the probiotic group. Probiotics may improve cognitive function by ameliorating peripheral inflammation and stress response [107]. SLAB51 is a novel probiotic formulation. Both in vivo and in vitro experiments have demonstrated its ability to modulate the BDNF pathway, increase the expression levels of neuroprotective proteins, and reduce the expression of neuronal death proteins. Another probiotic (De Simone Formulation) also exerted a protective role against POCD and resultant neuronal apoptosis and could block the BTG2/Bax axis by restoring the expression of miR-146a downregulated induced by anesthesia/surgery against POCD [108]. These results also underscore the neuroprotective effects of probiotics [109]. The perioperative use of oral probiotics to regulate gut microbiota homeostasis and reduce peripheral inflammation and stress responses can be a preventive measure for PNCDs.

As an indigestible food fiber, prebiotics can preferentially increase the growth and viability of probiotics such as *Lactobacillus* and *Bifidobacterium* and play an essential role in improving intestinal bacteria homeostasis. Different types of prebiotics include isomaltooligosaccharides, fructooligosaccharides, galactose oligosaccharides (GOS), soy oligosaccharides, and inulin. Although there have been a limited number of studies using prebiotics to treat PNCDs, prebiotics may play an important role in improving neurological function. Lactulose has been shown to have beneficial effects on neurological function recovery after stroke. This protective effect may be attributed to its ability to inhibit harmful bacterial proliferation and dysbiosis, repair damaged intestinal barriers, and reduce post-stroke inflammatory responses [110]. Some studies have also found that lactulose can not only repair intestinal barrier function but also regulate intestinal bacteria. It also increases the production of the beneficial intestinal metabolite SCFAs [111]. For example, another prebiotic, galactooligosaccharide-mixture (B-GOS), is a natural and highly stable dietary compound that promotes the proliferation of *Bifidobacteria* and certain *Lactobacilli*. Research has shown that B-GOS can inhibit the overactivation of microglial cells induced by anesthesia and surgery, thereby reducing neuroinflammation and cognitive function impairment [112]. When prebiotics (xylo-oligosaccharides, XOS) were fed perioperatively to APP/PS1 mice undergoing partial hepatectomy, the study found that XOS intervention effectively attenuated surgery-induced cognitive dysfunction and intestinal microbiota alteration, reduced inflammatory responses, and improved the integrity of tight junction barrier in the intestine and hippocampus [113]. The mechanism of the neuroprotective function of prebiotics may lie in the improvement of intestinal bacteria composition and intestinal barrier function, which produce a potential beneficial effect on the regulation of PNCDs affected by the gut-brain axis.

### Fecal Microbiota Transplantation

Fecal microbiota transplantation (FMT) is a method to transfer the complete intestinal bacteria or specific bacterial species from the donor to the intestine of the recipients, which can directly change the composition of the intestinal bacteria. The FMT can be used to observe the development of disease phenotypes and explore the role and mechanisms of gut microbiota in disease occurrence [11]. FMT is not only utilized for the treatment of gastrointestinal diseases such as constipation and diarrhea but is also increasingly used to treat neurological diseases. The application of FMT extends beyond gastrointestinal disorders like constipation and diarrhea to the treatment of neurological diseases. Research has revealed prominent differences in gut microbiota between elderly and young individuals. By performing FMT, transferring the gut microbiota from young mice to elderly mice can reverse the aging characteristics of the gut, eyes, and brain in elderly mice [114]. Studies also reveal that FMT therapy can mitigate depressive-like behaviors in stress-induced rat models, reduce abnormal activation of glial cells and astrocytes in the prefrontal cortex and hippocampus, and diminish IL-1β and TNF-α production [115]. For patients with Parkinson's disease, FMT seems to be promising in rebalancing gut microbiota, thus improving symptoms related to the autonomic nervous system [116]. Limited research exists on FMT for PNCDs. A study found that antibiotic-induced pseudo-germ-free mice exhibited abnormal behavior. However, transplanting the gut microbiota from control mice into pseudo-germ-free mice improved their abnormal behavior. These observations suggest that the diversity of the gut microbiota is beneficial for improving postoperative neurocognitive function [71]. Aged surgical mice received fecal microbiota transplantation from young or aged mice, and the authors found that surgery-induced cognitive impairment and alterations in the microbiota composition, and intestinal barrier disruption can be mitigated by transferring microbiota from young donors [117]. However, the application of FMT in humans has been encountered with challenges like the choice of transplantation methods, treatment efficacy, and the selection of suitable donor microbiota. More research is warranted to identify optimal FMT methods for treating PNCDs.

### Antibiotics

The role of antibiotics in modulating gut microbiota and their associated impact on cognitive function remains a subject of debate. In mice with chronic ethanol consumption-induced inflammation, using antibiotics to reduce gut microbiota complexity has been shown to improve neuroinflammation and intestinal inflammation [118]. However, in another study, it was reported that the use of ceftriaxone alone could disrupt the gut microbiota in mice, increasing the risk of cognitive impairment. Still, when administered as part of anesthesia/surgery procedures, antibiotics could mitigate postoperative memory and learning impairments in mice [69]. In a study using a pseudo-germ-free mouse model, the elimination of the gut microbiota in mice using a “cocktail” antibiotic regimen before anesthesia and surgery improved memory in elderly mice after surgery [13]. Perioperative treatment with cefazolin similarly attenuates the BBB disruptions and decreased levels of tight junction proteins, ZO-1 and Occludin in the mice of the POCD model. Cefazolin administration also reversed the changes in several fecal bacteria (β-, γ/δ-, ε-*Proteobacteria*, and *Bacteroidetes*) and the changes in expression of the two most abundant SCFAs (acetic and propionic acids) [119]. Overall, postoperative cognitive dysfunction may be attributed to an increase in the abundance of Gram-negative bacteria like *Escherichia coli *after disruption of gut homeostasis. Although antibiotics may disrupt the healthy gut microbiota in the host, the benefits of the clearance of the gut microbiota during the perioperative period seem to outweigh the potential risks.

### Other Microbiome-Targeted Therapies (MTTs)

In addition to antibiotics, the structure of the gut microbiota is influenced by external factors, with daily dietary intake being a significant contributor. Gut microbiota can synthesize various vitamins and fatty acids from the diet. As mentioned earlier, these metabolites can impact the nervous system by regulating factors such as BDNF and postsynaptic density protein. Therefore, improving dietary structure may be an additional effective measure in the treatment of cognitive impairment. Furthermore, exercise, being a cost-effective and widely applicable non-pharmacological therapy, has been shown to have significant potential in improving cognitive dysfunction [120]. Research has demonstrated that exercise can increase the abundance of beneficial bacteria, improve gut barrier integrity, inhibit host inflammatory responses indirectly, and reduce serum LPS levels in mice [121, 122]. A study of Chinese acupoint therapy found that using electroacupuncture to stimulate the acupoints Zusanli (ST36), Quchi (L111), Baihui (GV20), and Dazhui (GV14) 1 week before appendectomy in mice can modulate gut microbiota dysbiosis to ameliorate POCD; this may provide a novel target in POCD management [123].

These mechanisms highlight the potential of exercise in improving PNCDs. These are some possible mechanisms through which the gut microbiota may influence PNCDs, and exercise may hold promise as a practical approach to ameliorating PNCDs. Unfortunately, regarding the microbiome-targeted therapies described above, only a small number were conducted in a perioperative setting. Most human studies were conducted in the non-perioperative period, and an even smaller number were conducted in the perioperative setting. Based on these findings, microbiome-targeted therapies may be a potential new strategy for preventing PNCDs [124].

## Concluding Remarks

PNCDs are a common postoperative complication in elderly patients. In recent years, more and more studies have proved that gut microbiota plays a vital role in the development of the central nervous system and the homeostasis of nerve function, closely related to postoperative cognitive dysfunction. Disruption of the gut microbiota leads to increased intestinal vascular barrier permeability, which in turn results in the transmission of abnormal metabolites, neurotransmitters, and inflammatory mediators through the “gut-brain axis” to the central nervous system, causing changes in cognitive function. Current preclinical studies have confirmed that the perioperative use of prebiotics, probiotics, and antibiotics may help the host ameliorate the dysbiosis of the gut microbiota induced by anesthesia and surgery, thereby improving neurocognitive functioning. In addition, perioperative metabolites and anti-inflammatory properties have the potential for the prevention of PNCDs. Beyond these pharmacological options, transplantation of the gut microbiome from a healthy donor to a healthy recipient is also an effective therapy against PNCDs. Notably, therapeutic strategies such as electroacupuncture also have great potential in preventing and treating PNCDs. These results suggest that MTT may be a new strategy to prevent neurocognitive dysfunction.

In light of these findings, both animal and human studies suggest that intestinal microbiota dysbiosis is associated with an increased risk of PNCDs. However, the existing reports suggest promising research on the broad applications of gut microbiota, particularly in rodent models, there remains a scarcity of clinical research cohorts. We should prioritize further clinical research to validate the clinical utility of microbiome-targeted therapies and identify potential biomarkers for PNCDs. Besides the anti-inflammatory properties (probiotics, indolepropionic acid, dexamethasone, etc.) or treatments like FMT and EA described here, we call for more clinical trials to discover effective MTTs. Additionally, exploring the neuroregulatory mechanisms of gut microbiota may lead to the identification of therapeutic targets for systemic diseases and enhance diagnosis and prognosis assessment in this area.

## Data Availability

No datasets were generated or analyzed during the current study.
